# An Improved Extended Wavenumber Domain Imaging Algorithm for Ultra-High-Resolution Spotlight SAR

**DOI:** 10.3390/s25175599

**Published:** 2025-09-08

**Authors:** Gui Wang, Yao Gao, Weidong Yu

**Affiliations:** 1 Department of Space Microwave Remote Sensing System, Aerospace Information Research Institute, Chinese Academy of Sciences, Beijing 100094, China; wanggui16@mails.ucas.ac.cn; 2 School of Electronic, Electrical and Communication Engineering, University of Chinese Academy of Sciences, Beijing 100049, China; gaoyao18@mails.ucas.edu.cn; 3 National Key Laboratory of Microwave Imaging, Aerospace Information Research Institute, Chinese Academy of Sciences, Beijing 100094, China

**Keywords:** synthetic aperture radar, ultra-high-resolution, motion compensation, extended wavenumber domain algorithm

## Abstract

Ultra-high-resolution synthetic aperture radar (SAR) has important applications in military and civilian fields. However, the acquisition of high-resolution SAR imagery poses considerable processing challenges, including limitations in traditional slant range model precision, the spatial variation in equivalent velocity, spectral aliasing, and non-negligible error introduced by stop-and-go assumption. To this end, this paper proposes an improved extended wavenumber domain imaging algorithm for ultra-high-resolution SAR to systematically address the imaging quality degradation caused by these challenges. In the proposed algorithm, the one-step motion compensation method is employed to compensate for the errors caused by orbital curvature through range-dependent envelope shift interpolation and phase function correction. Then, the interpolation based on modified Stolt mapping is performed, thereby facilitating effective separation of the range and azimuth focusing. Finally, the residual range cell migration correction is applied to eliminate range position errors, followed by azimuth compression to achieve high-precision focusing. Both simulation and spaceborne data experiments are performed to verify the effectiveness of the proposed algorithm.

## 1. Introduction

Synthetic aperture radar (SAR) is a vital tool in remote sensing, experiencing exponential growth in recent decades [1,2,3]. Ultra-high-resolution SAR has been demonstrated to facilitate the detection of diminutive human-made targets (e.g., vehicles, boats, building details) and intricate surface structures (e.g., geologic faults, agricultural ridges) [4,5]. This capacity has resulted in the increase in its military and civilian application. However, the acquisition of ultra-high-resolution SAR imagery poses considerable processing challenges, including limitations in traditional slant range model precision, the spatial variation in equivalent velocity, spectral aliasing in the azimuth direction, and non-negligible error introduced by stop-and-go assumption. In recent years, a series of studies have been conducted to address these challenges, with a number of advances being made.

In terms of slant range models of SAR, traditional SAR systems generally used hyperbolic range model (HRM) [6]. However, HRM fails to precisely describe slant range history in long synthetic aperture or curved orbit scenarios, where equivalent velocity variation and high-order error terms significantly degrade model accuracy [7,8,9]. To this end, the equivalent acceleration model [7], modified equivalent squint range model [10], and the advanced equivalent squint range model [9] are proposed, which can accurately describe the range model with tolerable errors. To address the spatial variation in equivalent velocity caused by curved orbit, the sub-aperture coherent accumulation algorithm is proposed, with the core step of this algorithm including aperture segmentation, azimuth time-scale transformation, and high-order phase compensation [11]. In [12], a squint-equivalent acceleration range model is proposed to take the spaceborne squinted curved orbit into account precisely, and based on this new range model, a full-aperture imaging algorithm is proposed that can handle azimuthal variations in equivalent velocity and range variations in the two-dimensional frequency spectrum. In the domain of spectral aliasing, the cartesian decomposition backprojection (CFBP) algorithm applies spectral compression filters to lower the sampling rate and mitigate aliasing effects [13]. The two-step approach (TSA) addresses aliasing directly through azimuth convolution [14]. In [15], an efficient full-aperture approach that combines the TSA with autofocus based on the time-domain dealiasing is proposed for processing airborne spotlight SAR data. For large squint-angle imaging, the modified CFBP algorithm leverages coordinate transformation to optimize the range model, retaining the accuracy and efficiency advantages of the original method [16]. In addition, traditional spaceborne SAR processing usually uses the stop-and-go assumption; nevertheless, the stop-and-go assumption ignores satellite motion during signal transmission and reception, leading to severe phase errors and degraded pulse response functions in decimeter-level resolution or long integration time contexts [17,18]. To solve this problem, the compensation method in frequency and time domains is proposed in [5,18,19].

Beyond that, the one-step motion compensation (MOCO) algorithms proposed in [20,21] offer higher accuracy and computational efficiency by integrating all error compensation steps prior to range cell migration correction (RCMC). A hybrid strategy combining wavenumber-domain processing, aperture reduction, and backprojection achieves a balance between precision and computational load [22]. In addition, for space-variant errors induced by atmospheric turbulence, dedicated compensation schemes have notably improved focusing quality in ultra-high-resolution SAR system [23]. The extended wavenumber-domain algorithm (EWKA) integrates high focusing accuracy with robust motion compensation [24]. The numerical SAR method leverages numerical transfer functions to overcome constraints in extreme bistatic SAR configurations [25].

SAR imaging technology is advancing towards enhanced model precision, efficient compensation techniques, and scenario-specific algorithmic development [26,27]. For ultra-high-resolution SAR, higher-performance algorithms are required to achieve high focusing quality. This paper proposes an improved EWKA for ultra-high-resolution spaceborne spotlight SAR data processing. First, the curved orbit error is compensated using a one-step MOCO approach. Then, the improved Stolt mapping is applied to achieve the decoupling of range and azimuth, considering the effects of equivalent velocity variations. Then, the algorithm performs residual RCMC and azimuth fine focusing, completing the imaging processing. The algorithm demonstrates good focusing performance in both the range and azimuth directions through experimental verification.

This paper is organized as follows: Section 2 presents the imaging model, The proposed imaging algorithm is introduced in detail in Section 3. Then, Section 4 provides the simulation and spaceborne SAR data results, verifying the algorithm’s performance. Finally, Section 6 concludes the whole paper.

## 2. Imaging Model

In this section, the geometry of spotlight configuration and slant range model are both introduced. Then, the SAR echo model is also described in detail.

### 2.1. SAR Geometry

**Figure 1:**
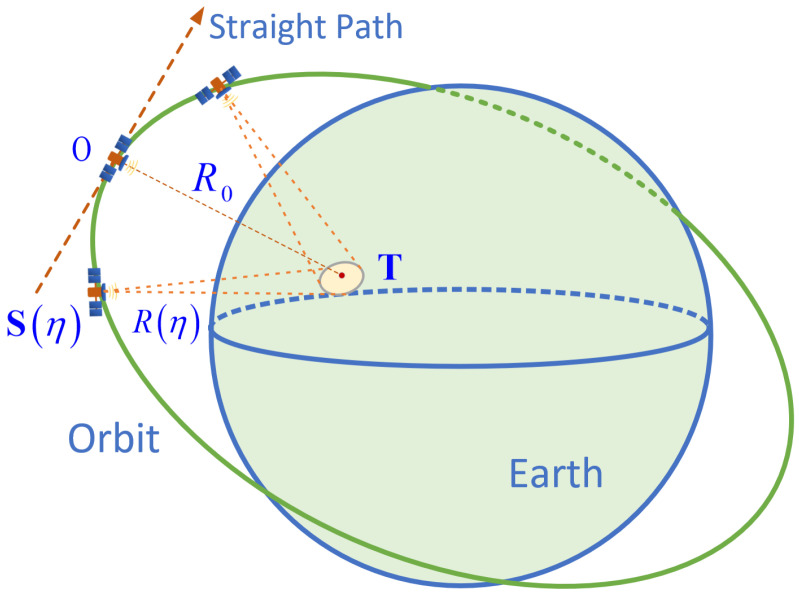
Geometric structure of spaceborne spotlight SAR imaging.

The geometric structure of spotlight SAR is illustrated in Figure 1. Assume the position of the target in the scene is T. In azimuth time η, the satellite position is S(η), and the echo delay $t_{0}\left(\eta \right)=2R\left(\eta \right)/c=2∥S(\eta )-T∥/c$ is a function of azimuth time, where *c* is the speed of light. The point *O* represents the satellite’s position at the zero-Doppler time. The dashed arrow represents the idealized linear motion path assumed in conventional SAR processing, whereas the green elliptical curve denotes the satellite’s actual curved trajectory. The yellow dashed line and circles indicate the continuous illumination of the target T by the radar beam during SAR data acquisition.

### 2.2. Slant Range Model

The satellite position S(η) and velocity in the Earth-centered inertial (ECI) frame can be determined from the six Keplerian orbital elements, which allow precise computation of the target center coordinates. The beam footprint is obtained by combining the satellite position with the beam pointing angle. Once the satellite position S(η) and target position T are represented in the coordinate system at each time step, the range slant range history $R\left(\eta \right)=∥S(\eta )-T∥$ can be calculated.

The slant range history of a point target can be approximated by the following equation in HRM [6]:

(1) $$R\left(\eta \right)\approx \sqrt{R_{0}^{2}+V_{eq}^{2}{\left(\eta -\eta_{0}\right)}^{2}}$$

where $\eta_{0}$ denotes the zero-Doppler time, i.e., the moment when the radar is closest to the target. $R_{0}$ is the minimum slant range at this moment, and $V_{eq}$. In roder to obtain $\eta_{0}$, $V_{eq}$, and $R_{0}$, $R^{2}\left(\eta \right)$ is expressed by the fitting model as follows:

(2) $$R^{2}\left(\eta \right)\approx p_{0}+p_{1}\eta +p_{2}\eta^{2}$$

where $p_{0}$, $p_{1}$, and $p_{2}$ are the coefficients of the fitting model. Note that this fitting is limited to the duration of the synthetic aperture, not the entire operation time. By comparing the above two expressions, the parameters of the HRM can be derived as

(3) $$\left\{\begin{array}{cc} \;V_{eq} & =\sqrt{p_{2}}\\ \eta_{0} & =-\frac{p_{1}}{2V_{eq}^{2}}=-\frac{p_{1}}{2p_{2}}\\ R_{0} & =\sqrt{p_{0}-\frac{p_{1}^{2}}{4p_{2}}} \end{array}\right.$$

**Figure 2:**
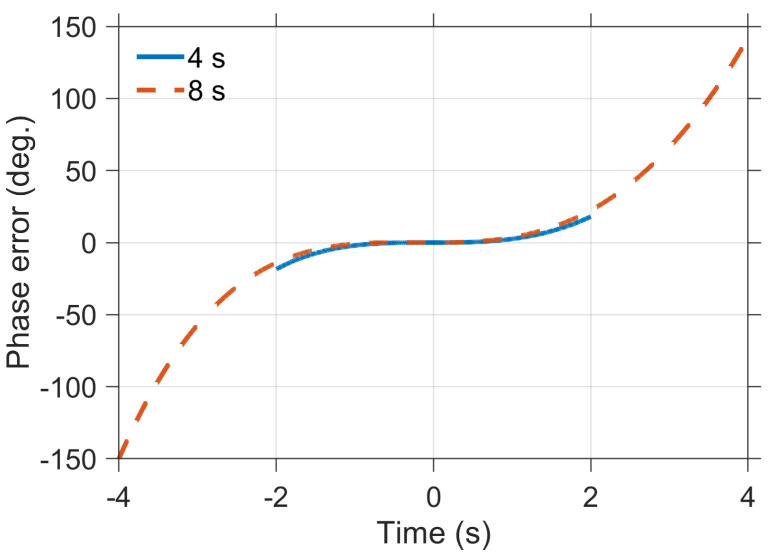
Phase error of HRM.

Here, the phase error introduced by HRM is simulated based on orbital parameters provided in Table 1. A pair of simulations are conducted with the synthetic aperture time of 4 s or 8 s. The carrier frequency is 9.65 GHz. The simulation results are shown in Figure 2. The equivalent velocity $V_{eq}$ is 7385.031 m/s, while the synthetic aperture time is 4 s. The equivalent velocity $V_{eq}$ is 7385.027 m/s, while the synthetic aperture time is 8 s. As shown in Figure 2, with the increase in synthetic aperture time, the corresponding phase error introduced by the HRM also increases. If the phase error exceeds 45°, image quality may be degraded. Therefore, in ultra-high-resolution spaceborne SAR imaging applications, the impact of orbital curvature and equivalent velocity variation must be fully considered, and more accurate imaging algorithms must be employed to effectively compensate for these errors.

### 2.3. SAR Echo Model

The echo signal of the point target T can be expressed as [6]

(4) $$\begin{array}{cc} S(\tau ,\eta ) & =w_{a}\left(\eta -\eta_{0}\right)w_{r}\left(\tau -t_{0}\left(\eta \right)\right)\exp\left\{j\pi K_{r}{\left(\tau -t_{0}\left(\eta \right)\right)}^{2}\right\}\exp\left\{-j2\pi f_{0}t_{0}\left(\eta \right)\right\}\\ \; & =w_{r}\left(\tau -\frac{2R(\eta )}{c}\right)w_{a}\left(\eta -\eta_{0}\right)\exp\left(-j\frac{4\pi f_{0}R\left(\eta \right)}{c}\right)\exp\left\{j\pi K_{r}{\left[\tau -\frac{2R(\eta )}{c}\right]}^{2}\right\} \end{array}$$

where $w_{a}\left(\;\cdot \;\right)$ represents the azimuth antenna pattern, $w_{r}\left(\;\cdot \;\right)$ represents the range window, and $f_{o}$ is carrier frequency. *c* is the speed of light, $f_{0}$ is the radar carrier frequency, $K_{r}$ is the range chirp rate, and τ is the range time. For simplicity, the range and azimuth envelope functions $w_{r}\left(\;\cdot \;\right)$ and $w_{a}\left(\;\cdot \;\right)$ are ignored in the following analysis.

## 3. Proposed Imaging Algorithm

**Figure 3:**
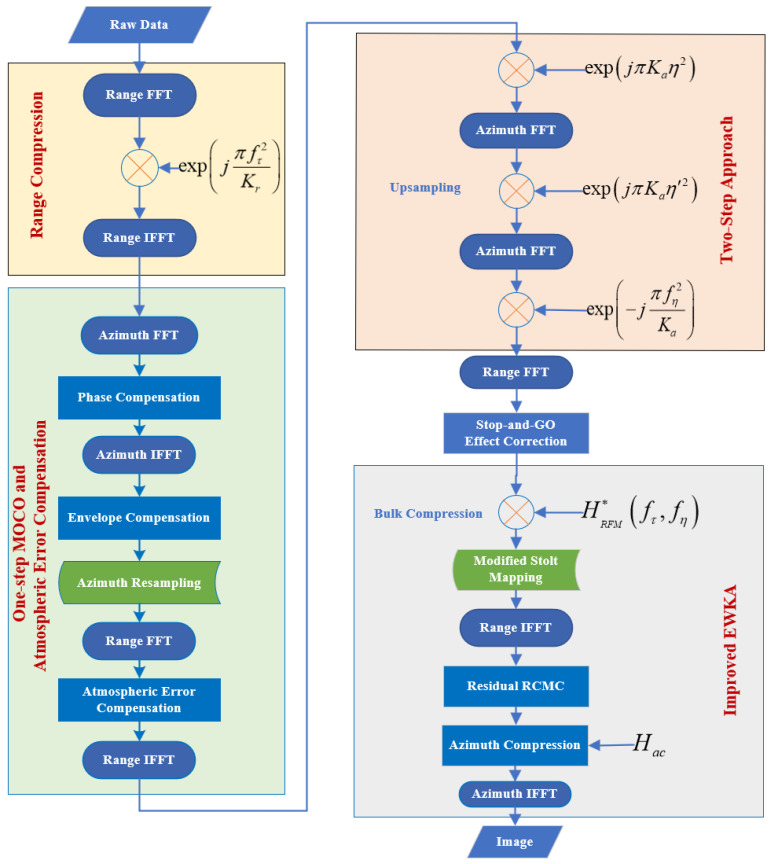
Flowchart of the proposed algorithm.

The flowchart (Figure 3) of the proposed improved EWKA illustrates the core processes of the algorithm. The proposed algorithm can be summarized as follows:

1. Range compression. It is achieved by applying a matched filter to the transmitted signal, which compresses the echo signal in the time domain.
2. One-step MOCO and atmospheric error compensation. This process employs phase function compensation along with range-dependent envelope migration interpolation to simultaneously address nonlinear slant-range variations resulting from the satellite’s curved trajectory, while also accounting for phase errors induced by atmospheric delays, as modeled by atmospheric error models.
3. Upsampling by using TSA. To address the azimuth aliasing issue caused by the large Doppler bandwidth, TSA is performed to obtain unaliasing signal in azimuth with high efficiency.
4. Stop-and-go effect correction. Errors introduced by the stop-and-go approximation are corrected to enhance the focusing quality.
5. Bulk compression and modified Stolt mapping. The modified Stolt mapping is applied to achieve the decoupling of range and azimuth.
6. Residual RCMC. Residual RCMC is performed using interpolation in the range-Doppler (RD) domain to correct the residual range migration errors caused by the variation in equivalent velocity with range.
7. Azimuth compression. The final azimuth compression is performed by considering the variation in equivalent velocity with range.

The aim of this flowchart is to precisely handle the errors caused by curved orbit and equivalent velocity variations by integrating steps such as one-step motion compensation, modified Stolt mapping, and residual RCMC, thereby achieving high-precision imaging of ultra-high-resolution spotlight SAR data. In the following subsection, each step of the proposed algorithm is described in detail.

### 3.1. Range Compression

The SAR echo is transformed into the frequency domain. Then, the matched filter of LFM signal can be written as

(5) $$H\left(f_{\tau }\right)=\mathrm{rect}\left(\frac{f_{\tau }}{|K_{r}|T_{r}}\right)\exp\left(j\pi \frac{f_{\tau }^{2}}{K_{r}}\right)$$

where rect(·) denotes the rectangular window function, $K_{r}$ is the chirp rate of the LFM signal, $T_{r}$ is the pulse duration, and $f_{\tau }$ represents the range frequency. After the matched filtering operation, the signal is transformed back to the range time domain via inverse fast Fourier transform (IFFT).

### 3.2. One-Step MOCO and Atmospheric Error Compensation

In SAR imaging, the radar platform typically moves along a curved orbit, which leads to nonlinear variations in the slant range history. To compensate for this nonlinear effect, a one-step MOCO method is adopted. The SAR echo signal is generally expressed as in Equation (4). To accurately compensate for range-dependent phase variations, range compression is first performed on the echo signal. Let $s_{1}\left(\tau ,\eta \right)$ denote the compressed signal, which can be written as

(6) $$s_{1}\left(\tau ,\eta \right)=\sin c\left(\tau -\frac{2R(\eta )}{c}\right)\exp\left(-j4\pi f_{0}\frac{R(\eta )}{c}\right)$$

This expression contains the main lobe of the compressed response and the phase term associated with the slant range. Next, the motion error is defined as the slant range difference between the actual curved trajectory of the radar platform and the ideal linear model. The motion error of the center target can be expressed as

(7) $$\Delta R_{c}\left(\eta \right)=R_{eq,c}\left(\eta \right)-\sqrt{R_{0}^{2}+V_{eq,c}^{2}\eta^{2}}$$

where $R_{eq,c}\left(\eta \right)$ and $V_{eq,c}$ denote the range history and the equivalent velocity of the center target in the scene. For each target along the range direction, the motion error is different. Suppose the motion error angle of the range time τ is ΔR(τ,η). Then, phase compensation is applied to the signal, resulting in the compensated signal $s_{2}\left(\tau ,\eta \right)$.

(8) $$s_{2}\left(\tau ,\eta \right)=s_{1}\left(\tau ,\eta \right)\;\cdot \;\exp\left(j4\pi f_{0}\frac{\Delta R(\tau ,\eta )}{c}\right)$$

The signal then becomes

(9) $$s_{2}\left(\tau ,\eta \right)=\sin c\left(\tau -\frac{2R(\eta )}{c}\right)\;\cdot \;\exp\left(-j4\pi f_{0}\frac{R_{\mathrm{HRM}}\left(\eta \right)}{c}\right)$$

Here, $R_{\mathrm{HRM}}\left(\eta \right)=R\left(\eta \right)-\Delta R\left(\eta \right)$ represents the phase-corrected slant range under HRM. After phase compensation, envelope shift due to spatially variant error still exists. To compensate for envelope shift, an interpolation-based resampling method is applied to obtain the final compensated signal $s_{3}\left(\tau ,\eta \right)$.

(10) $$s_{3}\left(\tau ,\eta \right)=\sum_{l}\sin c\left(\frac{2\Delta R(\tau ,\eta )}{c}F_{r}-l\right)\;\cdot \;s_{2}\left(\tau +\frac{l}{F_{r}},\eta \right)$$

where Fr is the sampling frequency in range. Through the dual compensation of phase and envelope, the spatially variant error in the signal is effectively suppressed. The final signal expression becomes

(11) $$s_{3}\left(\tau ,\eta \right)=\sin c\left(\tau -\frac{2R_{\mathrm{HRM}}\left(\tau ,\eta \right)}{c}\right)\;\cdot \;\exp\left(-j4\pi f_{0}\frac{R_{\mathrm{HRM}}\left(\tau ,\eta \right)}{c}\right)$$

This expression demonstrates that after one-step MOCO processing, the spatially variant errors have been corrected, enabling the subsequent use of linear trajectory model-based processing. In low-resolution modes, the azimuth scan angle is small, and the slant-range errors introduced by the atmosphere can be approximated as constant, having limited impact on the imaging. However, in spotlight modes, where the synthetic aperture time is long and the scan angle is large, atmospheric effects vary significantly with different angles. This leads to changes in slant-range errors, causing target defocusing. The error model is given by [18]

(12) $$\Delta R_{\mathrm{tropo}}\left(\eta \right)=\frac{\Delta R_{tropo}^{ref}}{\cos\alpha_{i}\left(\eta \right)}$$

where $\Delta R_{tropo}^{ref}$ represents the reference slant-range error, and $\cos\alpha_{i}\left(\eta \right)$ is the azimuth angle at a specific azimuth time.

At large azimuth angles, the slant-range and phase errors increase significantly. Therefore, targeted compensation using atmospheric models and real-time angles is required to ensure imaging quality. Additionally, factors such as electromagnetic layer dispersion and DEM (digital elevation model) terrain mismatches can also impact SAR imaging, especially for long integration times and high-resolution imaging. These effects usually require correction with meteorological and DEM data to ensure imaging quality, though these issues are not the primary focus of this paper and are only briefly mentioned here.

### 3.3. Upsampling by Using TSA

In spotlight SAR imaging, azimuth frequency aliasing occurs because the azimuth signal bandwidth is typically higher than the pulse repetition frequency (PRF). To address this, TSA is proposed, which addresses aliasing directly through azimuth convolution [14]. The azimuth frequency modulation rate $K_{a}$ can be expressed as

(13) $$K_{a}=\frac{2V_{eq}^{2}}{\lambda R_{0}}$$

Then the azimuth dechirping operation is applied to the echo signal s(τ,η):

(14) $$s^{\prime }\left(\tau ,\eta \right)=s\left(\tau ,\eta \right){*}_{az}\exp\left(j\pi K_{a}\eta^{2}\right)$$

where ${*}_{az}$ denotes the azimuth-direction convolution operation. This step is equivalent to performing a matched filter between the original echo signal and the reference frequency-modulated signal, which results in spectral compression and prevents azimuth frequency aliasing. This convolution process can also be efficiently performed using the fast Fourier transform (FFT), as follows:

(15) $$s^{\prime }\left(\tau ,\eta^{\prime }\right)=h_{2}\left(\eta^{\prime }\right)\;\cdot \;F_{a}\left[s\left(\tau ,\eta \right)\;\cdot \;h_{1}\left(\eta \right)\right]$$

where $h_{1}\left(\eta \right)=\exp\left(j\pi K_{a}\eta^{2}\right)$ is the reference signal used for dechirping, and $h_{2}\left(\eta^{\prime }\right)=\exp\left(j\pi K_{a}{\left(\eta^{\prime }\right)}^{2}\right)$ is the residual phase function used to recover the signal structure. $F_{a}\left[\;\cdot \;\right]$ denotes the azimuth direction FFT, and $\eta^{\prime }$ is the upsampled azimuth time, defined as

(16) $$\eta^{\prime }=\frac{n\;\cdot \;PRF}{K_{a}P},\;n=-\frac{P}{2}+1,\ldots ,\frac{P}{2}$$

Since the number of output points after upsampling $P>N_{a}$ (the number of input azimuth points), zero padding is required. The upsampling factor, determined by $K_{a}$, PRF, and *P*, can be flexibly chosen to suit different FFT implementation strategies. After dechirping processing, the frequency-domain spectrum of the signal has changed. To ensure compatibility with subsequent imaging algorithms, a frequency-domain modulation recovery step is required. This process can be accomplished by multiplying by the following modulation function:

(17) $$H\left(f_{\eta }\right)=\exp\left(-j\frac{\pi f_{\eta }^{2}}{K_{a}}\right)$$

where $f_{\eta }$ is the azimuth frequency. This operation effectively reconstructs the original frequency spectrum, ensuring that subsequent strip-map imaging algorithms can correctly focus the image.

### 3.4. Stop-and-Go Effect Correction

In traditional SAR echo modeling, the stop-and-go approximation is commonly used, which ignores the motion of the radar platform during signal transmission and reception. This assumption is quite reasonable for airborne SAR systems. For example, when the slant range at the scene center is 20 km and the flight speed is 200 m/s, the platform moves approximately 0.03 m during the signal round-trip time, which has a negligible effect. However, for low-Earth-orbit spaceborne SAR systems, the platform speed can reach approximately 7100 m/s. If the scene center slant range is 800 km, the satellite can move around 38 m during the signal transmission period. This error significantly affects image quality in ultra-high-resolution imaging and must be compensated. The errors caused by the stop-and-go approximation primarily consist of two effects: the slow-time effect and the fast-time effect, whose errors should be compensated in the imaging processing.

The slow-time effect mainly causes target position shifts in the azimuth direction but does not severely impact the focusing quality. This error can be corrected in the RD domain by multiplying the following phase compensation function [5]:

(18) $$H_{\mathrm{slow}}\left(f_{\eta },R_{0}\right)=\exp\left(j2\pi \frac{R_{0}}{c}f_{\eta }\right)$$

The fast-time effect causes a mismatch in the range frequency spectrum, introducing phase errors in the two-dimensional frequency domain, which affect image focusing. This error can be corrected using the following two-dimensional frequency-domain compensation function [5,17,18]:

(19) $$H_{\mathrm{fast}}\left(f_{\tau },f_{\eta }\right)=\exp\left(j2\pi \frac{f_{\eta }}{K_{r}}f_{\tau }\right)$$

By jointly compensating for these two effects, the errors introduced by the stop-and-go approximation can be effectively eliminated, improving the imaging accuracy and positioning precision of spaceborne spotlight SAR systems.

### 3.5. Bulk Compression and Modified Stolt Mapping

After one-step MOCO, the slant-range history between the radar and the target can be modeled using the HRM, as shown in Equation (1). After applying a two-dimensional Fourier transform to the echo signal of a point target, its expression in the two-dimensional frequency domain becomes [6]

(20) $$S_{2df}\left(f_{\tau },f_{\eta }\right)=W_{r}\left(f_{\tau }\right)W_{a}\left(f_{\eta }\right)\exp\left(-j\frac{4\pi R}{c}\sqrt{{\left(f_{0}+f_{\tau }\right)}^{2}-\frac{c^{2}f_{\eta }^{2}}{4V_{eq}^{2}}}\right)$$

where $W_{r}\left(f_{\tau }\right)$ and $W_{a}\left(f_{\eta }\right)$ are the spectral envelope functions in the range and azimuth directions, respectively. The conjugate spectrum of the reference point target can be expressed as

(21) $$H_{RFM}\left(f_{\tau },f_{\eta }\right)=\mathrm{conj}\left[S_{2df}\left(f_{\tau },f_{\eta };R_{ref}\right)\right]$$

where $R_{ref}$ is the reference slant range. Multiplying the original signal by the reference function yields the coarsely focused frequency-domain signal:

(22) $$S_{RFM}\left(f_{\tau },f_{\eta }\right)=W_{r}\left(f_{\tau }\right)W_{a}\left(f_{\eta }\right)\exp\left(-j\frac{4\pi (R-R_{ref})}{c}\sqrt{{\left(f_{0}+f_{\tau }\right)}^{2}-\frac{c^{2}f_{\eta }^{2}}{4V_{eq}^{2}}}\right)$$

In the improved Stolt interpolation, with azimuth velocity $V_{eq}=V_{eq,c}$ assumed constant, the signal is mapped from $\left(f_{\tau },f_{\eta }\right)$ to a new domain $\left(f_{\tau }^{\prime },f_{\eta }\right)$ to decouple range and azimuth for fine focusing, where $f_{\tau }^{\prime }$ denotes the new range frequency. The modified Stolt mapping is defined as

(23) $$f_{\tau }^{\prime }=\sqrt{{\left(f_{0}+f_{\tau }\right)}^{2}-\frac{c^{2}f_{\eta }^{2}}{4V_{eq}^{2}}}-\sqrt{f_{0}^{2}-\frac{c^{2}f_{\eta }^{2}}{4V_{eq}^{2}}}-f_{0}$$

The interpolated signal is expressed as

(24) $$S_{RFM}^{\prime }\left(f_{\tau }^{\prime },f_{\eta }\right)=W_{r}\left(f_{\tau }^{\prime }\right)W_{a}\left(f_{\eta }\right)\exp\left(-j\frac{4\pi (R-R_{ref})}{c}\left[f_{0}\left(1+D(f_{\eta },V_{eq})\right)+f_{\tau }^{\prime }\right]\right)$$

where the function $D(f_{\eta },V_{eq})$ is defined as

(25) $$D\left(f_{\eta },V_{eq}\right)=\sqrt{1-\frac{\lambda^{2}f_{\eta }^{2}}{4V_{eq}^{2}}}.$$

### 3.6. Residual RCMC

Performing an inverse Fourier transform along the range frequency yields

(26) $$S_{rd}\left(\tau ,f_{\eta }\right)=\sin c\left(\tau -\frac{2(R_{rd}\left(f_{\eta }\right)+\Delta R_{cm})}{c}\right)\;\cdot \;W_{a}\left(f_{\eta }\right)\;\cdot \;\exp\left(-j\frac{4\pi \;\cdot \;\left(R-R_{ref}\right)D\left(f_{\eta },V_{eq}\right)f_{0}}{c}\right)$$

where $R_{rd}\left(f_{\eta }\right)$ denotes the linear range-dependent term in the RD domain, and $\Delta R_{cm}$ is the residual RCMC error caused by equivalent velocity variation, given by

(27) $$\Delta R_{cm}\left(f_{\eta }\right)=\frac{R_{ref}}{D(f_{\eta },V_{eq,R})}-\frac{R_{ref}}{D(f_{\eta },V_{eq,c})}$$

where $V_{eq,R}$ denotes the actual equivalent velocity along the range direction. In the RD domain, residual RCMC is performed through interpolation. The interpolation function can be expressed as

(28) $$S\left(\tau ,f_{\eta }\right)=\sum_{l}\sin c\left(\frac{2\Delta R_{cm}\left(f_{\eta }\right)}{c}F_{r}-l\right)\;\cdot \;S_{rd}\left(\tau +\frac{l}{F_{r}},f_{\eta }\right)$$

This operation resamples the signal to the corrected range position, achieving precise RCMC.

These steps play a crucial role in establishing the connection between actual equivalent velocity $V_{eq,R}$ and equivalent velocity $V_{eq,c}$ in processing. By introducing $D(f_{\eta },V_{eq,R})$, and $D(f_{\eta },V_{eq,c})$ into the signal model, the discrepancy between the actual and assumed velocity is explicitly characterized, which in turn provides the basis for performing RCMC.

### 3.7. Azimuth Compression

Azimuth compression is performed as the final step of the imaging processing. The matched filter is defined as

(29) $$H_{\mathrm{ac}}\left(f_{\eta }\right)=\exp\left(j\frac{4\pi \;\cdot \;\left(R-R_{ref}\right)\;\cdot \;\left(D\left(f_{\eta },V_{eq,R}\right)-1\right)}{\lambda }\right)$$

Finally, an inverse Fourier transform along the azimuth frequency converts the signal back to the azimuth time domain, resulting in the focused image:

(30) $$I\left(\tau ,\eta \right)=\sin c\left(\tau -\frac{2(R-R_{ref})}{c}\right)\;\cdot \;\sin c\left(\eta \right)$$

## 4. Experiments

In this section, the simulation and spaceborne SAR data experiments are both performed to verify the effectiveness of the proposed algorithm.

### 4.1. Simulation Experiment

**Figure 4:**
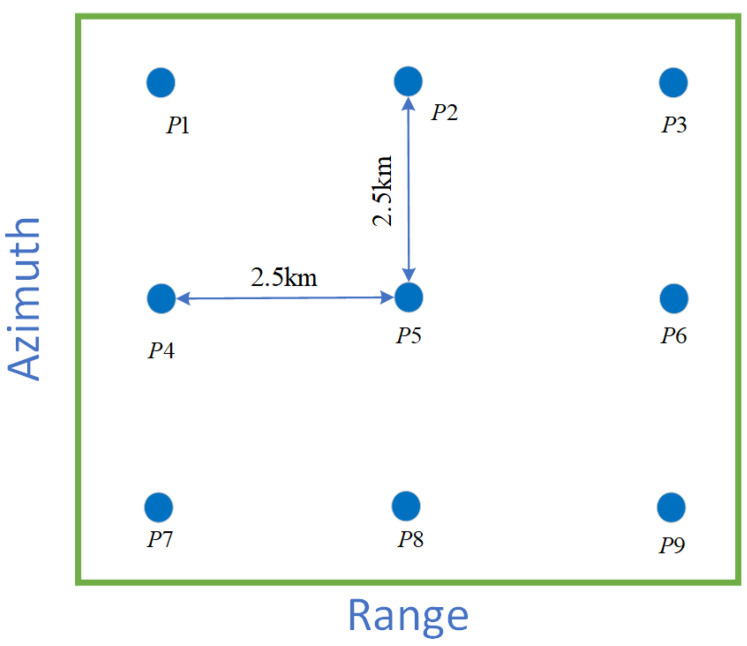
Location of the 9 point targets.

The point target simulation is conducted with orbital parameters specified in Table 2. The raw echo signal is generated by incorporating the satellite’s motion during both transmission and reception, employing an LFM signal with a 10 GHz carrier frequency, 1.2 GHz range bandwidth, and 10 µs pulse duration. A 3×3 array of point targets is symmetrically distributed at the edges and center as a 5km×5km scene (Figure 4), where the system is designed to achieve a theoretical range resolution of 0.108 m and an azimuth resolution of 0.150 m. SAR system parameters are summarized in Table 3.

**Figure 5:**
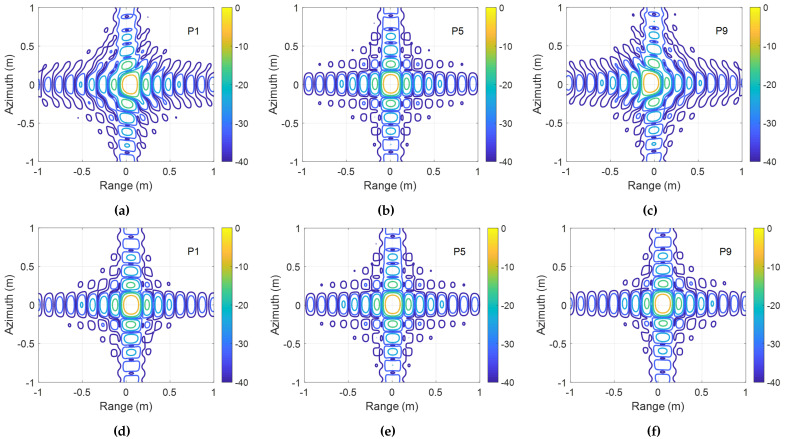
Contour plots of targets P1, P5, and P9. (**a**–**c**) Using the conventional EWKA. (**d**–**f**) Using the proposed algorithm.

The simulation results are shown in Figure 5, displaying the contour plots of the responses of targets P1, P5, and P9. Panels (a)–(c) correspond to the conventional EWKA [24] (without compensating for equivalent velocity variations and residual RCMC in azimuth focusing after Stolt mapping), while panels (d)–(f) show results from the proposed algorithm (Figure 1). While the central point P5 is well focused by both methods, P1 and P9 exhibit significant focusing errors in (a) and (c), whereas (d) and (f) achieve high-quality focusing. Table 4 quantifies this performance via the impulse response width (IRW), peak sidelobe ratio (PSLR), and integrated sidelobe ratio (ISLR), confirming that the proposed algorithm matches theoretical resolution and maintains acceptable metric ranges.

**Figure 6:**
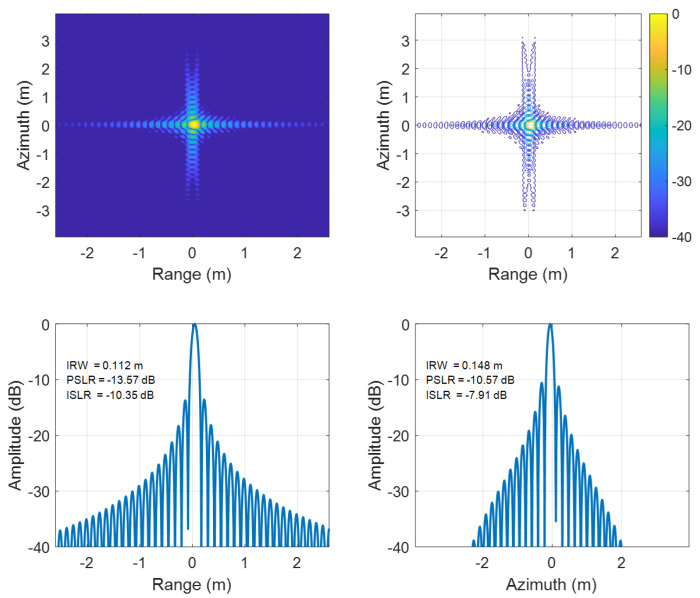
Results of targets P1 using the conventional EWKA.

**Figure 7:**
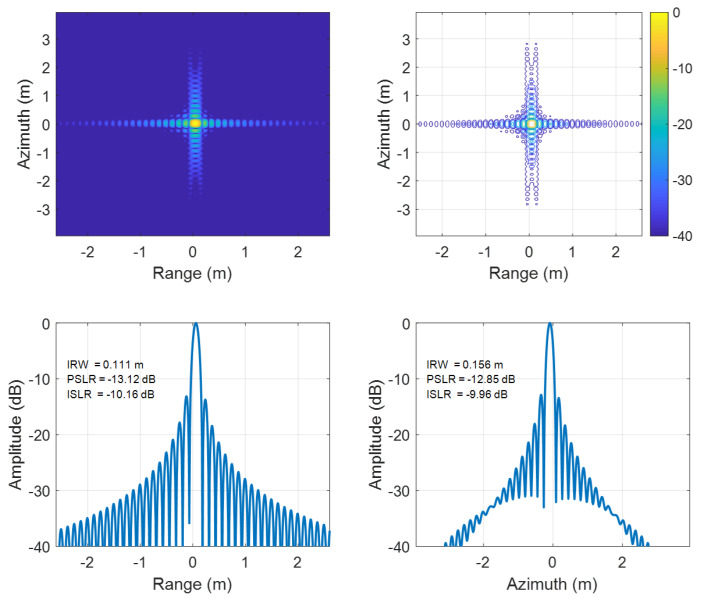
Results of targets P1 using the proposed algorithm.

**Figure 8:**
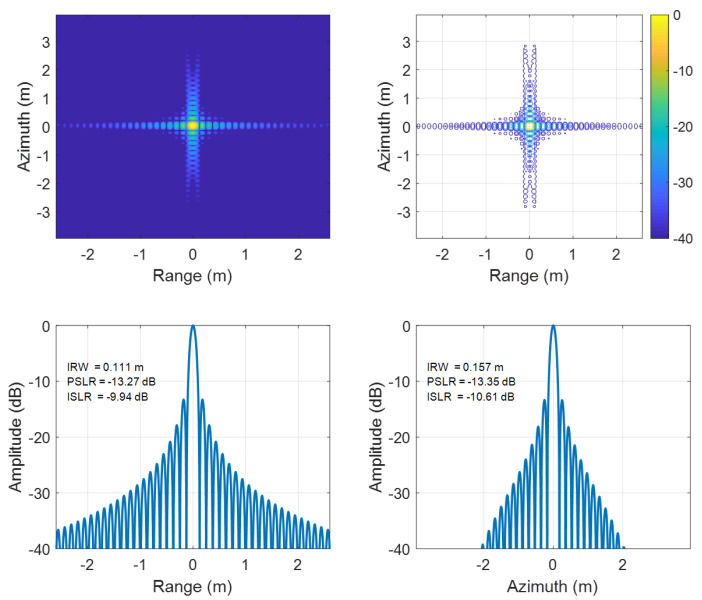
Results of targets P5 using the conventional EWKA.

**Figure 9:**
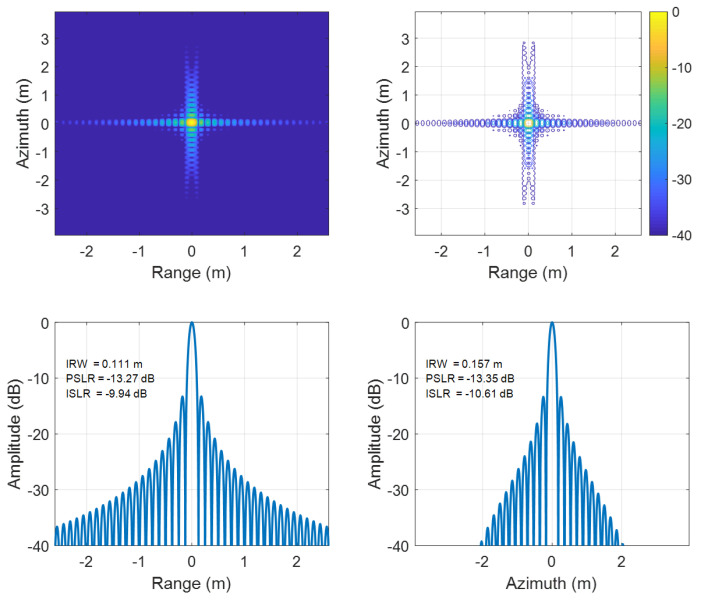
Results of targets P5 using the proposed algorithm.

To further contrast edge and central performance, detailed comparisons at the edge point P1 (Figure 6 and Figure 7) and central point P5 (Figure 8 and Figure 9) reveal that the traditional EWKA has drastically worse ISLR at the edge (P1), though differences are minimal at the center (P5). This indicates pronounced defocusing of edge target in the traditional method, whereas the proposed algorithm achieves superior edge focusing, validating its effectiveness in both range and azimuth directions.

**Figure 10:**
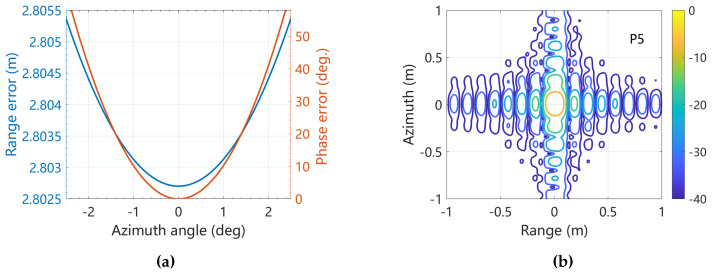
(**a**) Atmospheric error; (**b**) imaging result of P5 without atmospheric error compensation.

In order to illustrate the impact of atmospheric error, a typical error scenario was simulated. The parameter $\Delta R_{tropo}^{ref}$ was set to 2.80 m. The resulting error is depicted in Figure 10a. If the atmospheric error remains uncorrected, it can reach up to 50° at the edge. The imaging result for point P5 without atmospheric error compensation is shown in Figure 10b, where it is evident that the error is substantial and significantly degrades the image quality.

### 4.2. Spaceborne SAR Data Experiment

To further validate the effectiveness of the proposed algorithm, this subsection presents experimental verification using raw spotlight mode SAR data obtained from the Gaofen-3 (GF-3) satellite. The parameters of the raw data are listed in Table 5. The scene covers a multi-scene area including roads, buildings (urban zone), farmland (suburban), and ships (coastal waters), with the scene center at $31.958^{\circ }N$ and $118.612^{\circ }E$ (Nanjing, China). The acquisition time was 09:30 (local time) on March 11, 2017. The platform-track parameters were satellite speed 7539.35m/s and beam speed 6745.31m/s.

The system parameters are as follows: the carrier frequency is 5.4GHz, the look angle is $33.75^{\circ }$, and the incidence angle is $38.51^{\circ }$. The range bandwidth is 240MHz, with a corresponding range sampling frequency of 266.67MHz. The pulse duration of the transmitted signal is 45μs, and the pulse repetition frequency is 3742.80Hz. For the azimuth channel, the azimuth bandwidth is 19380Hz, the azimuth steering range is $\pm 1.78^{\circ }$, and the synthetic aperture time is 8.58s. Theoretical azimuth and ground range resolutions are 0.40m and 0.89m, respectively.

**Figure 11:**
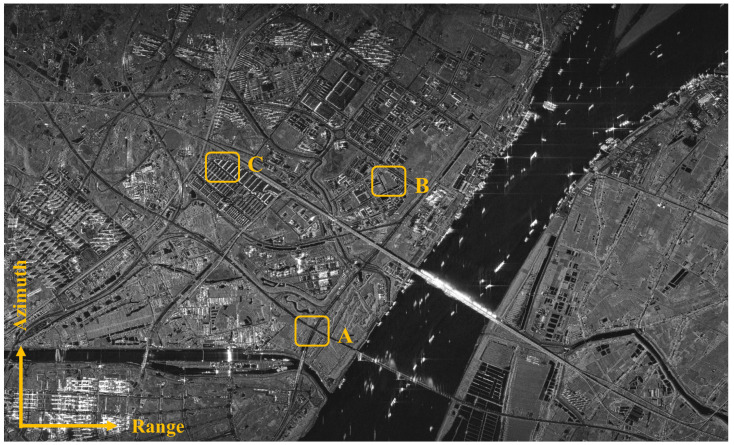
The imaging result of GF-3 spotlight data by using the proposed algorithm.

**Figure 12:**
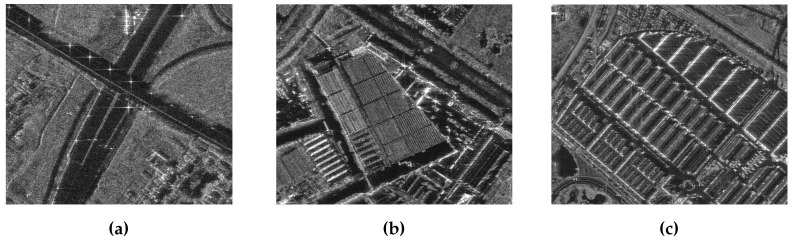
(**a**), (**b**), and (**c**) Extracted zoomed-in views, which are captured from parts A, B, and C in Figure 11, respectively.

The imaging result obtained from processing the GF-3 spotlight data using the proposed algorithm is shown in Figure 11, which exhibits good focusing quality. Figure 12 is updated to include zoomed-in comparative images of three scenes: (A) crossroads, (B) farmland, and (C) buildings, processed by the proposed algorithm, showing clear road edges, sharp farmland ridges, and distinct building contours. The imaging results demonstrate that the proposed algorithm performs well in various scenarios and ensures that the experiment can be reproduced.

## 5. Discussion

The proposed algorithm integrates one-step MOCO, atmospheric error compensation, stop-and-go model error correction, modified Stolt mapping, and residual RCMC. Although its computational load is higher than that of the traditional EKWA method, it still operates in the frequency domain, with a computational complexity of $O(N^{2}\log N)$. Here, *N* denotes the number of sampling points in both the range and azimuth directions of the echo data, which are assumed to be the same. In contrast to the backprojection (BP) algorithm, which has a complexity of $O(N^{3})$, the proposed approach still offers significantly higher computational efficiency.

Furthermore, we discuss the application scenarios of the algorithm. In our simulation experiments, the scene swath was set to 5 km with a resolution of approximately 0.1 m, indicating that the algorithm is capable of achieving decimeter-level imaging. However, for resolutions finer than 0.1 m, it may be challenging to maintain a swath width of 5 km. This is primarily because the bulk compression step utilizes the equivalent velocity $V_{eq,c}$ at the scene center for processing. The phase error introduced by this approximation can be expressed as

(31) $$\theta_{error}\left(f_{\tau },f_{\eta }\right)=\frac{4\pi (R-R_{ref})}{c}\left(\sqrt{{\left(f_{0}+f_{\tau }\right)}^{2}-\frac{c^{2}f_{\eta }^{2}}{4V_{eq,R}^{2}}}-\sqrt{{\left(f_{0}+f_{\tau }\right)}^{2}-\frac{c^{2}f_{\eta }^{2}}{4V_{eq,c}^{2}}}\right)$$

**Figure 13:**
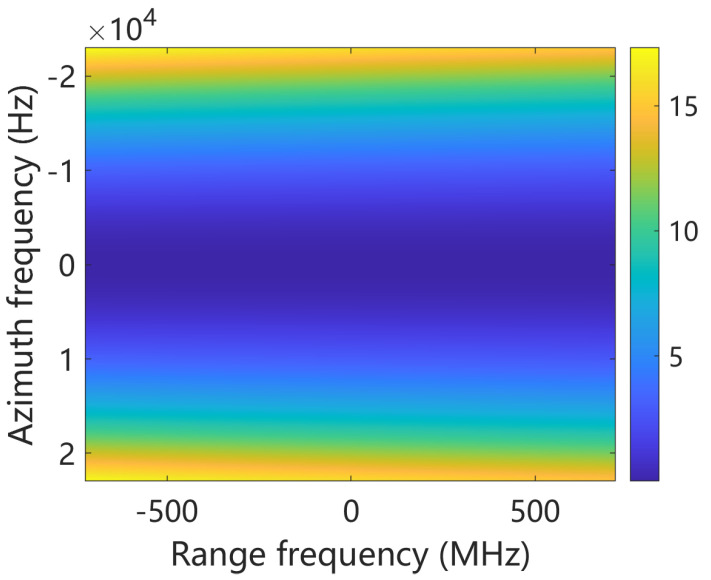
Phase error introduced by bulk compression in 2D frequency domain.

In the point target simulation, the phase error $\theta_{error}$ for the edge target P1 is illustrated in Figure 13. The equivalent velocity difference between P1 and P5 is approximately 0.63 m/s, resulting in a maximum phase error $\theta_{error}$ variation of 17°. Although this error is acceptable in the current experiment, it may become non-negligible in higher-resolution applications (below 0.1 m), where the equivalent velocity varies more significantly across the scene. To address this issue for ultra-high-resolution imaging, one feasible solution is to divide the imaging area into sub-blocks, process each block individually, and then mosaic the sub-images into a complete final image.

## 6. Conclusions

This paper proposes a novel imaging algorithm for ultra-high-resolution spotlight SAR. A one-step MOCO technique is first employed to correct errors induced by the curved orbit. Subsequently, azimuth decompression processing is applied to precisely compensate for residual RCMC and azimuth focusing errors arising from the range dependency of the equivalent velocity. Simulation and experimental results validate the superior focusing performance of the proposed algorithm. Future research efforts will focus on optimizing the trade-off between computational efficiency and imaging accuracy, as well as developing universal processing frameworks for multistatic and distributed SAR systems.

## Figures and Tables

**Table 1:** Orbit parameters.

Parameter	Value
Eccentricity	0.0011
Inclination	$97.67^{\circ }$
Semi-major axis	6890.22 km
Argument of perigee	$68.54^{\circ }$
Ascending node	$140.37^{\circ }$

**Table 2:** Orbit parameters.

Parameter	Value
Eccentricity	0.0012
Inclination	$97^{\circ }$
Semi-major axis	7000 km
Argument of perigee	$90^{\circ }$
Ascending node	$0^{\circ }$

**Table 3:** Spotlight SAR simulation parameters.

Parameter	Value
Carrier frequency	10 GHz
Range bandwidth	1.2 GHz
Range sampling frequency	1.4 GHz
Pulse duration	10 µs
Azimuth resolution	0.150 m
Range resolution	0.108 m
Ground range scene size	5 km
Azimuth scene size	5 km

**Table 4:** Image quality of the focused targets.

	Range			Azimuth		
	IRW (m)	PSLR (dB)	ISLR (dB)	IRW (m)	PSLR (dB)	ISLR (dB)
P1 (EWKA)	0.112	−13.57	−10.35	0.148	−10.57	−7.91
P1 (Prop.)	0.111	−13.12	−10.16	0.156	−12.85	−9.96
P5 (EWKA)	0.111	−13.27	−9.94	0.157	−13.35	−10.61
P5 (Prop.)	0.111	−13.27	−9.94	0.157	−13.35	−10.61
P9 (EWKA)	0.112	−13.48	−10.18	0.147	−10.26	−7.37
P9 (Prop.)	0.112	−13.16	−10.38	0.159	−13.58	−10.88

**Table 5:** Main parameters of GF-3 data in spotlight mode.

Parameter	Value
Carrier frequency	5.4 GHz
Look angle	33.75°
Incidence angle	38.51°
Range bandwidth	240 MHz
Range sampling frequency	266.67 MHz
Pulse duration	45 μs
PRF	3742.80 Hz
Azimuth bandwidth	19380 Hz
Azimuth steering range	±1.78°
Synthetic aperture time	8.58 s
Satellite speed	7539.35 m/s
Beam speed	6745.31 m/s
Scene location	31.958° N, 118.612° E
Acquisition time	09:30 on 11 March 2017

## Data Availability

The original contributions presented in this study are included in this article. Further inquiries can be directed to the corresponding author.

## Abbreviations

The following abbreviations are used in this manuscript:

CFBP	cartesian decomposition backprojection
DEM	digital elevation model
EWKA	extended wavenumber domain algorithm
FFT	fast Fourier transform
IFFT	inverse fast Fourier transform
HRM	hyperbolic range model
IRW	impulse response width
ISLR	integrated sidelobe ratio
MOCO	motion compensation
PRF	pulse repetition frequency
PSLR	peak sidelobe ratio
RCMC	range cell migration correction
RD	range-Doppler
SAR	synthetic aperture radar
TSA	two-step approach
